# Higher return to pre-injury type of sports after revision anterior ligament reconstruction with lateral extra-articular tenodesis compared to without lateral extra-articular tenodesis

**DOI:** 10.1007/s00167-022-07018-y

**Published:** 2022-06-23

**Authors:** Michèle N. J. Keizer, Reinoud W. Brouwer, Feike de Graaff, Roy A. G. Hoogeslag

**Affiliations:** 1 Center for Human Movement Sciences, University of Groningen, University Medical Center Groningen, UMCG Sector F, FA 23, PO Box 219, 9713 AV Groningen, The Netherlands; 2 Department of Orthopedic Surgery, Martini Hospital, Groningen, The Netherlands; 3 Centre for Orthopaedic Surgery and Sports Medicine OCON, Hengelo, The Netherlands

**Keywords:** ACL, Revision, Lateral extra-articular tenodesis, Return to sport

## Abstract

**Purpose:**

To evaluate the rate of return to pre-injury type of sports (RTS type) in patients after revision anterior cruciate ligament reconstruction (ACLR) with lateral extra-articular tenodesis (LET) compared to patients after revision ACLR without LET.

**Methods:**

Seventy-eight patients who underwent revision ACLR with an autologous ipsilateral bone-patellar tendon-bone autograft with and without LET were included at least one year after surgery (mean follow-up: 43.9, SD: 29.2 months). All patients filled in a questionnaire about RTS type, the Knee injury and Osteoarthritis Outcome Score (KOOS), the International Knee Documentation Committee subjective form (IKDC_subjective_), and the Tegner activity score.

**Results:**

The RTS type for revision ACLR with LET was 22 of 42 (52%), whereas 11 of 36 (31%) of the patients who underwent revision ACLR without LET returned to the pre-injury type of sport (*p* = 0.05). No significant differences were found in KOOS subscores, IKDC_subjective_, and Tegner activity scores.

**Conclusion:**

An additional LET increases the rate of RTS type after revision ACLR.

**Level of evidence:**

III.

## Introduction

Return to sports rate after revision anterior cruciate ligament (ACL) reconstruction (ACLR) is low; about half of the patients after revision ACLR are reported to return to their pre-injury type of sports (RTS type) [11, 22]. One of the reasons for the low RTS type might be the presence of residual rotational laxity after ACLR. Because injury to structures in the anterolateral corner (ALC) of the knee is reported to result in a decrease in rotational stability [15], an increasing interest in several extra-articular surgical procedures to stabilize the ALC has been seen in the past years [1, 4], and it has been reported that residual rotational laxity after ACLR decreased when this was combined with ALC reconstruction [2, 7, 13]. Moreover, reduced failure rate, increased subjective outcome scores, anterior tibial translation, and tibial internal rotation have been reported after ACLR combined with ALC reconstruction [2, 3, 12, 16, 17]. Indicators for this procedure seem to be young age (which might be a proxy for activity level [24]), participation in pivoting sports, and chronic ACL injury [18].

One of these extra-articular ALC reconstruction techniques is a lateral extra-articular tenodesis with the iliotibial band (LET) [14]. However, evidence of whether revision ACLR with additional LET increases RTS compared to revision ACLR without LET is lacking. It is expected that patients after an ACLR with high rotational stability of the knee are more likely to RTS type compared to those with low rotational stability. Due to the expected lower rotational laxity after a LET, the RTS type might be higher after ACLR with LET than without LET.

The present study aimed to evaluate the RTS type in patients after revision ACLR combined with LET compared to RTS type in patients after revision ACLR without LET. Moreover, functional outcome scores were evaluated after ACLR with and without LET. It is hypothesized that patients who underwent a combined revision ACLR with LET show higher RTS type and better functional outcome scores than patients who underwent revision ACLR without LET.

## Materials and methods

A retrospective cohort study was conducted at the Centre for Orthopaedic Surgery and Sports Medicine OCON in Hengelo, The Netherlands. The institutional review board (IRB) of OCON Centre for Orthopaedic Surgery and Sports Medicine approved this study (IRB nr: 2020101).

Seventy-eight patients were included for analyses (mean follow-up: 43.9, SD: 29.2, range follow-up 12–192 months; Table 1). Patients who underwent revision ACLR with an autologous ipsilateral bone-patellar tendon-bone (BPTB) between February 2012 and February 2020 and with a minimum follow-up of 1 year were eligible to participate in this study. Exclusion criteria were a history of contralateral ACL injury and re-rupture of the revision ACLR. After inclusion, patients were distributed into two groups: (1) patients who underwent revision ACLR combined with LET (ACLR_LET group), and (2) patients who underwent revision ACLR without LET (ACLR group).

**Table 1:** Baseline characteristics of the patients^a^

	ACLR_LET group	ACLR group	*P* value
Number of patients	42	36	
Woman/man	9/33	12/24	n.s
Age [mean (SD)]	27.6 (7.6)	31.3 (8.9)	n.s
Left/right knee	19/23	18/18	n.s
Months between revision and participation [mean (SD)]	30.7 (12.9)	62.4 (35.3)	< 0.00
Primary graft used			n.s
Hamstrings autograft	42	39	
Level of sports before primary injury			n.s
Recreational	4	3	
Competition regional	35	28	
Competition national	4	6	
Cartilage injury			n.s
Medial	10	15	
Lateral	9	10	
Patellar	6	3	
Meniscal injury medial			n.s
No	25	13	
Yes, no treatment	1	1	
Yes, meniscectomy	13	18	
Yes, meniscal repair	3	4	
Meniscal injury lateral			n.s
No	21	23	
Yes, no treatment	7	1	
Yes, meniscectomy	13	12	
Yes, meniscal repair	1	0	

*n.s.* not significant

^a^*ACLR* group patients who underwent revision ACLR without LET, *ACLR_LET* group patients who underwent revision ACLR combined with LET

The primary outcome measure was the RTS type (returned/not returned). Secondary outcome measures were: the Dutch versions of the Knee injury and Osteoarthritis Outcome Score (KOOS) [6], the International Knee Documentation Committee subjective form (IKDC_subjective_) [8], and the Tegner activity score [20].

### Procedure

Together with an explanation of the study, an online questionnaire including homemade open questions about the RTS type [11] (Table 2), the KOOS, IKDC_subjective_, and Tegner activity score was sent to patients by e-mail. By submitting the questionnaire, the patients gave their informed consent. After the patients had filled in the questionnaire, the researcher extracted baseline characteristics from the patient’s file and information about the presence or absence of concomitant cartilaginous or meniscal injuries and the graft used for primary ACLR from the patients’ operative form.

**Table 2:** Homemade questions about return to sports (translated form Dutch)

What kind of sport(s) did you perform before your knee injury?
At what level did you perform these sport(s)?
Did you perform the same sport(s) again after your first ACLR?
If so, at what level did you perform your sport(s)?
Did you perform the same sport(s) again after revision ACLR?
If so, at which level did you perform your sport(s)?
If you did not return to the same sport(s), what was the reason?

### Surgical technique

One experienced orthopaedic surgeon (RAGH) performed all surgeries. One-stage or two-stage ACL revision surgery was performed. All ACL revisions were performed using a bone-patellar tendon-bone (BPTB) autograft, which was harvested from the ipsilateral leg. The ACL graft was fixed with interference screws at 20 degrees of knee flexion. LET was performed with a modified deep Lemaire technique as previously described [10, 14] and was fixated with an interference screw (RCI; Smith and Nephew) [9].

### Statistical analysis

The data were processed and statistical testing was performed using SPSS version 26 (IBM SPSS Statistics for Windows, Version 27.0. Armonk, NY: IBM Corp). A Chi-square test was used to compare the distribution of RTS type (returned/not returned) between groups. As the criteria for an independent sample t-test were not met (kurtosis > 1.96), Mann–Whitney *U* tests were used to compare the KOOS subscales, IKDC_subjective_, and Tegner activity score between groups. Moreover, statistical tests were performed to check for differences in baseline characteristics between groups. An alpha level of *p* ≤ 0.05 was considered statistically significant.

A post-hoc power calculation using proportions of 0.524 and 0.306, an alfa of 0.05, and group sizes of 36 and 42, revealed a power of 50% for analysis regarding RTS type calculated using G*Power 3.1.

## Results

### Primary outcome: RTS type

A significant RTS type was found between the ACLR_LET (52.4% RTS) and ACLR group (30.6% RTS; X(1) = 3.78, *p* = 0.05). The level of return to sports is presented in Table 3.

**Table 3:** Level of sports resumption

	ACLR_LET group	ACLR group
Same	13	7
Lower	9	4
Not	20	25

^a^*ACLR* group patients who underwent revision ACLR without LET, *ACLR_LET* group patients who underwent revision ACLR combined with LET

### ***Secondary outcomes: KOOS, IKDC***_***subjective***_***, KOOS, and Tegner activity score***

No significant differences were found between the ACLR_LET and ACLR groups in KOOS subscores, IKDC_subjective_ scale, and Tegner activity score (Table 4).

**Table 4:** Subjective outcomes between groups ^a^

	ACLR_LET group	ACLR group	*p* value (2-tailed)
KOOS_symptoms_ [median (range)]	60.7 (35.7–89.3)	60.7 (17.9–100)	n.s
KOOS_pain_ [median (range)]	91.7 (52.8–100)	94.4 (38.9–100)	n.s
KOOS_ADL_ [median (range)]	98.5 (61.8–100)	98.5 (30.9–100)	n.s
KOOS_sport_ [median (range)]	75 (20–100)	70 (0–100)	n.s
KOOS_qol_ [median (range)]	53.1 (18.8–75)	56.3 (25–1000)	n.s
IKDC_subjective_ [median (range)]	81.7 (47.6–95.1)	81.1 (17.1–95.1)	n.s
Tegner activity score [median (range)]	6 (1–10)	6 (2–10)	n.s

^a^*KOOS* The knee injury and osteoarthritis outcome score, *IKDC*_*subjective*_ the International Knee Documentation Committee subjective form

*n.s.* not significant

## Discussion

The most important finding of the present study is that more patients returned to their pre-injury type of sports after revision ACLR combined with an additional LET than patients who underwent revision ACLR without additional LET.

One possible explanation that patients with an additional LET are more likely to return to their pre-injury type of sports may be that the laxity of the knee is reduced after this procedure [17]. After ACLR without LET, rotational laxity is reported to be higher than after ACLR with LET [21]. One cadaver and two experimental studies using a hamstring tendon for primary ACLR combined with an extra-articular lateral reconstruction procedure indeed showed that the internal rotational laxity of the knee was lower compared to ACLR without extra-articular lateral reconstruction [7, 17]. Lower rotational laxity will increase the stability of the knee and may increase the trust in the knee and, therefore, may increase RTS probabilities. Moreover, in cadavers, untreated ALC injuries, together with untreated meniscal tears and collateral ligament tears, could lead to an overload of the ACL (graft) and consequently higher changes to graft failure [23]. Indeed, previous studies showed that after (primary) ACLR with extra-articular lateral reconstruction, the failure rate is considerably decreased, suggesting higher stability of the knee [2, 5, 19].

In line with the current study, Lee et al. [13] also found a higher return to sports level after revision ACLR combined with an extra-articular lateral reconstruction procedure using allograft tendons for ACLR compared to ACLR without extra-articular lateral reconstruction. In contrast with the current study, they also found higher IKDC and Tegner activity score after the combined procedure. A higher IKDC and Tegner activity score and higher KOOSsymptom and KOOSsport scores were also found in other studies [2, 7]. No significant differences was found between groups in the IKDC score, KOOS subscores, or Tegner activity score. The differences in findings may be because of differences in graft choice: Lee et al. [13] used allografts, Alm et al. [2] used hamstring, BPTB and quadriceps autografts, whereas in the present study only BPTB autografts were used. Patients who are operated using specific types of grafts, such as allografts and hamstring tendons, for ACLR might benefit more from an extra-articular lateral reconstruction procedure. The absence of significant differences between groups in IKDC, Tegner activity score, and KOOS scores in the current study might also be due to the low number of patients in the current study (power < 13%).

Besides the retrospective nature of the present study, there are some other limitations. No objective instrumented assessments such as passive rotational knee laxity were used to measure knee function. Another limitation is the significant difference in time between participation and surgery between the ACLR_LET (30.7 months) and ACL group (62.4 months). This significant difference is due to the transition of surgical technique from revision ACLR without to revision ACLR with LET. This may have influenced the RTS type between the two groups. However, a subgroup analysis where the groups had equal time between surgery and participation could not be performed as the number of participants would have been too small. Moreover, no subgroup analysis with a follow-up of 2 years was performed. This is because the power is too small for this analysis and approximately the same percentage of the patients included in our study with a follow-up of 1 year returned to sports (42%) compared to the patients with a follow-up of two years (45.6%). In addition, the sample size of this study was low and, therefore, our results should be interpreted as an indication and not as definite.

The clinical relevance of the present study is that it provides evidence for using an additional LET in combination with a revision ACLR with BPTP as RTS is higher after ACLR with LET than ACLR without LET.

## Conclusion

A combined LET with revision ACLR in patients after failure of a primary ACLR increases the rate of return to the pre-injury type of sports compared to revision ACLR without LET. Therefore, an additional LET combined with a revision ACLR is beneficial for those patients and should be considered by orthopaedic surgeons.
